# Uterine lumen fluid is metabolically semi-autonomous

**DOI:** 10.1038/s42003-022-03134-0

**Published:** 2022-03-01

**Authors:** Constantine A. Simintiras, Jessica N. Drum, Hongyu Liu, M. Sofia Ortega, Thomas E. Spencer

**Affiliations:** 1 Division of Animal Science, University of Missouri, Columbia, MO 65211 USA; 2 Division of Obstetrics, Gynecology, and Women’s Health, University of Missouri, Columbia, MO 65201 USA

**Keywords:** Reproductive biology, Metabolomics

## Abstract

Uterine lumen fluid (ULF) is central to successful pregnancy establishment and maintenance, and impacts offspring wellbeing into adulthood. The current dogma is that ULF composition is primarily governed by endometrial glandular epithelial cell secretions and influenced by progesterone. To investigate the hypothesis that ULF is metabolically semi-autonomous, ULF was obtained from cyclic heifers, and aliquots incubated for various durations prior to analysis by untargeted semi-quantitative metabolomic profiling. Metabolite flux was observed in these ULF isolates, supporting the idea that the biochemical makeup of ULF is semi-autonomously dynamic due to enzyme activities. Subsequent integrative analyses of these, and existing, data predict the specific reactions underpinning this phenomenon. These findings enhance our understanding of the mechanisms leading to pregnancy establishment, with implications for improving fertility and pregnancy outcomes in domestic animals as well as women.

## Abstract (web summary)

Uterine lumen fluid (ULF) is metabolically semi-autonomous in that biochemical reactions are active within ULF independently of external parameters. This finding enhances the understanding of the mechanisms leading to pregnancy establishment.

## Introduction

Infertility affects 10–15% of couples^1^ with approximately 14% of confirmed pregnancies lost within the first 8 weeks^2^; however, our understanding of pregnancy establishment remains poor as ~38% of female-factor subfertility is diagnosed as unexplained in women^3^. The inadequate endometrial function is presumed etiologic for two-thirds of implantation failures^4^. In addition to pregnancy loss, perturbations in the uterine environment can result in chronic adult–onset health impairments for the offspring, in line with the developmental origins of the health and disease paradigm^5^. Female subfertility is also prevalent in cattle, with calving rates often below 30% in dairy cows^6^. Also, most unsuccessful pregnancies fail within the first 21 days post-insemination^7^—the etiology of many of which is a uterine defect, as opposed to paternal or embryo (e.g., aneuploidy) causes^8^. Of relevance, the epithelial lining of the uterine endometrium, particularly the glands, secretes and selectively transports a variety of substances into the lumen of the uterus that is essential for pregnancy establishment via effects on embryo survival, growth, and implantation^9^. Consequently, a better understanding of uterine lumen fluid (ULF) formation and composition regulation is essential to improving fertility and the well-being of subsequent generations.

Conceptus (embryo and extra-embryonic tissues) elongation in ruminants is unique and coincides with a period of high pregnancy loss^7^. The process is characterized by: (i) a morphological transition from spherical to ovoid to tubular to filamentous; (ii) a rapid increase in trophectoderm length, involving cell proliferation that is essential for sufficient production of the maternal pregnancy recognition signal, interferon tau^10^; and (iii) onset of extraembryonic membrane differentiation^11–13^. In cattle, elongation commences around Day 12 and continues through Day 16, which is the day of maternal pregnancy recognition^11^. Metabolism is presumed central to conceptus elongation as (i) many genes expressed by the elongating conceptus pertain to metabolism^14,15^; (ii) greater enrichment of differentially expressed genes relating to metabolism was observed in long vs. short (i.e., developmentally incompetent) conceptuses^16,17^; (iii) progesterone (P4) acts via the endometrium to amplify select endometrial metabolites in ULF by Day 14^18^; and (iv) metabolically related enzymes dominate proteins in the ULF by Day 15. Indeed, the largest protein function categories of the Day 16 ULF proteome are “metabolic processes” and “catalytic activity”^19^.

Bovine^18–20^ and human^21^ ULF are highly dynamic and contain metabolically relevant enzymes. Accordingly, our central hypothesis is that ULF is metabolically semi-autonomous, i.e. that select biochemical pathways are active within ULF due to enzymatic activity and occur independently of external influences^22^. This phenomenon has been observed in cerebrospinal fluid (CSF)—an interstitial fluid, like ULF—in which the tryptophan–kynurenine pathway is active^23^. Furthermore, imbalances in CSF tryptophan, kynurenine, kynurenic acid, and quinolinic acid levels are associated with neurodegenerative disorders, such as multiple sclerosis, schizophrenia, and Alzheimer’s disease^23^. As such, it is tempting to suggest that the metabolic semi-autonomy of CSF may be a physiologically relevant phenomenon.

To test whether ULF is also metabolically semi-autonomous, ULF from cattle was sampled, aliquoted, and incubated for various durations, prior to comprehensive metabolomic analysis. The resulting data support the hypothesis that ULF is metabolically semi-autonomous. Moreover, the semi-autonomous reactions in the ULF involve metabolites associated with conceptus development, as well as metabolites previously identified in the ULF of highly fertile animals—cumulatively suggestive of this phenomenon being physiologically significant.

## Results and discussion

**Fig. 1:**
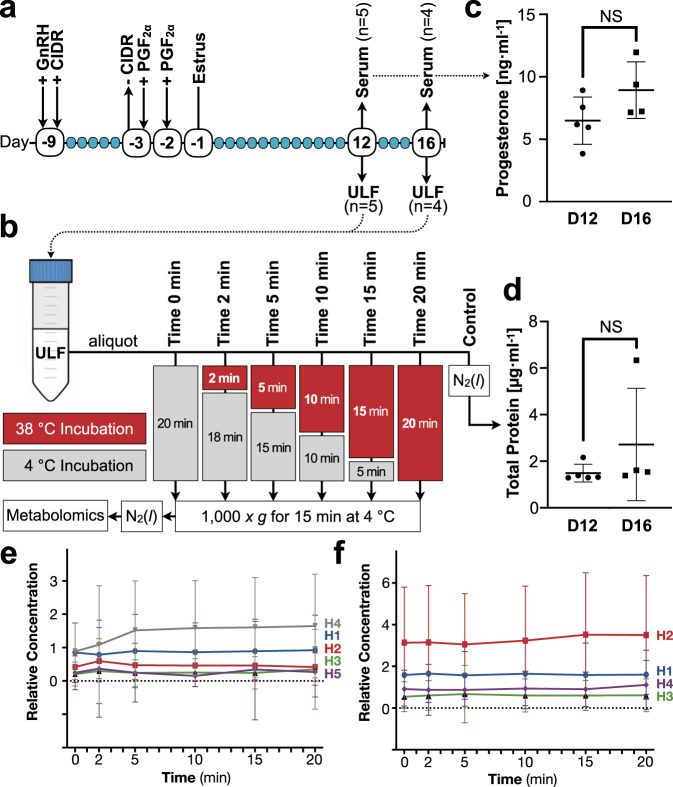
Experimental design depiction and validation. **a** The estrous cycles of nine heifers (H) were synchronized by administration of: gonadotropin-releasing hormone (GnRH); controlled internal drug release (CIDR) intra-vaginal progesterone insert; and prostaglandin F2α (PGF2α). Uterine lumen fluid (ULF), and serum, were recovered on Days 12 and 16. **b** Recovered ULF was aliquoted and select aliquots were incubated for various durations prior to centrifugation and supernatant storage until analysis. **c** Mean (horizontal line ± S.D.) serum progesterone levels of heifers from which ULF was obtained, at the time of isolation, on Day 12 (D12; H1-5; *n* = 5) or 16 (D16; H1-4; *n* = 4). **d** Mean (horizontal line ± S.D.) total protein concentration of control ULF lavage aliquots on D12 (*n* = 5) and D16 (*n* = 4). **e**, **f** Mean (±S.E.M.) relative concentration of all ULF metabolites on D12 (*n* = 317); **e** and D16 (*n* = 324); **f** from each heifer throughout the ULF incubation period. No effect of time or heifer was observed. Additional abbreviations: not significant (NS).

To test our hypothesis that ULF is metabolically semi-autonomous, the estrous cycles of nine dairy heifers were synchronized, and ULF was sampled using a non-surgical approach on Days 12 and 16 by uterine lavage using phosphate-buffered saline (PBS) (Fig. 1a). The recovered ULF was immediately aliquoted, incubated for various durations, and then comprehensively analyzed by high-throughput untargeted semi-quantitative metabolomics (Fig. 1b). Cyclic (non-pregnant) animals were selected as the conceptus secretes many metabolites^13^ and enzymes^24^. Thus, sampling ULF from cyclic animals allowed us to interrogate our hypothesis in ULF free from compounding conceptus-derived factors.

Mean (±S.D.) serum P4 at the time of ULF lavage of Day 12 and 16 cohorts was 6.5 ± 1.9 ng ml^−1^ and 8.9 ± 2.3 ng ml^−1^, respectively (Fig. 1c); correspondingly, mean (±S.D.) *corpus luteum* diameters were 22.6 ± 2.7 mm (Day 12) and 20.5 ± 1.9 mm (Day 16), cumulatively confirming successful estrous cycle synchronization. Moreover, total protein was measured in control ULF aliquots (Fig. 1b), and the mean (±S.D.) across all heifers was 2.0 ± 1.6 µg ml^−1^ (Fig. 1d). As the total volume of fluid in the uterine lumen is approximated to be 200 µl in cattle^25^—and extrapolating that the 10 ml PBS flush conducted here resulted in a 50-fold dilution—we can assume that the ULF retrieved here, undiluted, comprised 100 µg ml^−1^ protein. For reference, single mammalian cells contain about 200 μg ml^−1^ protein^26^. Thus, the total protein concentration of ULF observed is orders of magnitude lower than what would be expected from mass cell lysis. Further, the total concentration of all metabolites in the ULF of each heifer on Days 12 (Fig. 1e) and 16 (Fig. 1f) remained unchanged during incubation, further confirming that any exogenous metabolite consumption or release was negligible at worst, and absent at best.

**Fig. 2:**
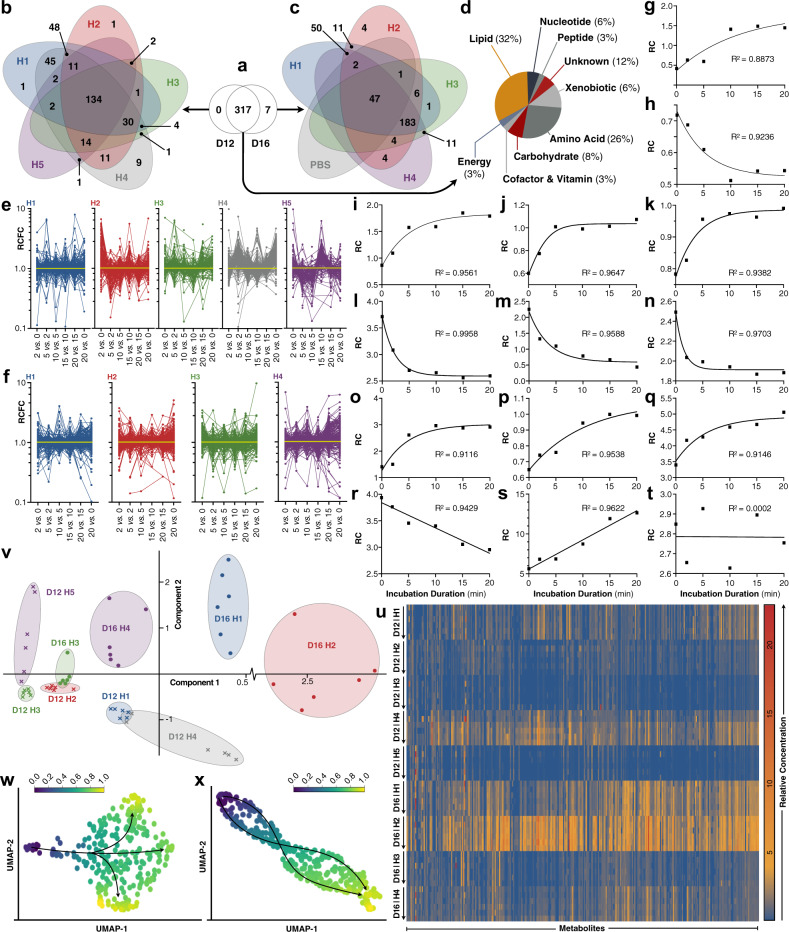
Uterine lumen fluid is metabolically semi-autonomous. **a** Venn diagram of the number of metabolites present in the uterine lumen fluid (ULF) of heifers on Day 12 (D12) vs. 16 (D16). **b**, **c** Breakdown of the number of metabolites identified, or not, in the ULF of each heifer (H) on **b** Day 12 (H1–5), and **c** Day 16 (H1–4), in addition to the phosphate-buffered saline (PBS) lavage control. **d** Super-pathway distribution of the metabolites identified in ULF on both days (*n* = 317). **e**, **f** Relative concentration fold-changes (RCFC) of all individual metabolites in Day 12 (**e**) and Day 16 (**f**) ULF of each heifer (H1–H5) between incubation intervals. **g**–**t** Examples of specific individual metabolite flux [relative concentrations (RC)] over time within single uteri, with corresponding trendlines and coefficients of determination (*R*^2^). Specifically: **g** 3-hydroxy-3-methylglutarate (D12/H3); **h** 3-hydroxy-3-methylglutarate (D12/H2); **i** 5-oxoproline (D12/H3); **j** fructose (D12/H4); **k** glycerophosphocholine (D12/H1); **l** nicotinamide mononucleotide (D12/H1); **m** spermidine (D12/H1); **n** phenylacetylglycine (D12/H1); **o** arginine (D16/H4); **p** sphingosine (D16/H1); **q** putrescine (D16/H1); **r** putrescine (D16/H2); **s** urate (D16/H2); and **t** N-acetylglycine (D12/H1). **u** Heatmap of individual metabolite relative concentration variation across heifers. Arrows correspond to incubation timepoints in ascending order. Shades of blue, yellow, and red correspond to metabolite relative concentrations, whereas green cells denote undetected metabolites. **v** Landmark Principal Component Analysis of ULF from all heifers. Each point represents the metabolomic profile of ULF at a single incubation time-point. **w**, **x** Pseudo-time analysis of the **w** Day 12 and **x** Day 16 ULF metabolome from all heifers at all incubation intervals. Additional abbreviation: uniform manifold approximation and projection unit (UMAP).

A total of 317 metabolites were detected in ULF on Day 12, with just 7 additional metabolites identified on Day 16 (Fig. 2a), despite 134 (Fig. 2b) vs. 230 (Fig. 2c) metabolites common to the ULF of all subjects on each respective day. The majority of these 317 metabolites clustered under lipid and amino acid metabolism (Fig. 2d), similarly to ULF from beef heifers on Days 12–14^18^. Regarding the hypothesis, metabolite flux was observed in ULF on Days 12 (Fig. 2e) and 16 (Fig. 2f), confirming that the biochemical makeup of ULF is indeed semi-autonomously dynamic. In fact, observed individual metabolite flux increases include: 15.5-fold (diethanolamine); 13.2-fold (N-palmitoyl-sphingosine); 9.8-fold (spermine); 9.6-fold (mannitol/sorbitol); and 7.4-fold (butyrylcarnitine) (Supplementary Data 1). Specific examples of multi-directional individual metabolite levels over time are presented in Fig. 2g–t.

Interestingly, individual metabolite concentrations (Fig. 2u), as well as flux (Fig. 2e, f), were relatively inconsistent across subjects, as cumulatively summarized by Fig. 2v. Specific examples include 3-hydroxy-3-methylglutarate—which displayed an increasing trend in the ULF lavage of one subject on Day 12 (Fig. 2g), yet the opposite trajectory in a different Day 12 subject (Fig. 2h). Similarly, putrescine levels rose in a Day 16 ULF lavage (Fig. 2q) and fell during the incubation of another lavage from a different Day 16 subject (Fig. 2r).

Two inevitable aetiologies likely underly this heterogeneity: (i) biological variation between animals (inter-subject variation; Fig. 2u); and (ii) asynchrony (intra-subject variation). The latter may be partially reflected by serum P4 level variation (Fig. 1c). It is worth noting that achieving perfect estrous cycle synchrony is impossible^27^—in other words, we suspect that, had we lavaged these uteri just 5 min sooner or later, the trends presented in Fig. 2g–t may look quite different. Nonetheless, despite this inevitable heterogeneity, these data demonstrate that ULF is, in fact, a semi-independently dynamic microenvironment, and, therefore, metabolically semi-autonomous, as hypothesized.

To identify potential commonalities among these data, bioinformatic pseudotime analyses were performed. More specifically, given that single uterine lavages were obtained from each subject, these metabolomic data are inherently cross-sectional, thus lacking biological temporal specificity and resolution. Thus, to partially circumvent this, trajectorial inference, or pseudotime, analyses, were performed to map this high-dimensional, cross-sectional, and asynchronous data (Supplementary Data 1 and 2) to a series of one-dimensional quantities (pseudotimes), for measuring any relative progression in the absence of explicit time series data^28^. The results suggest that three divergent pathways exist in the ULF of Day 12 subjects (Fig. 2w), compared to two, seemingly functionally redundant, pathways on Day 16 (Fig. 2x).

**Fig. 3:**
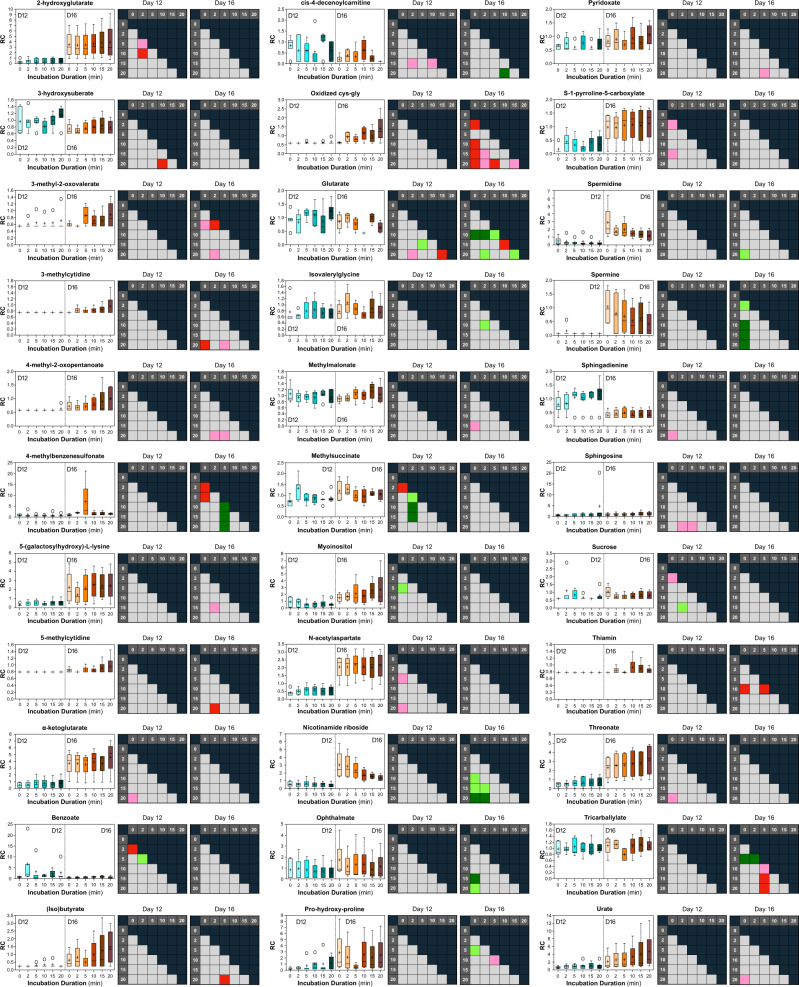
Qualitative and semi-quantitative interrogation of uterine lumen fluid metabolic semi-autonomy. Metabolites exhibiting statistically significant (*P* ≤ 0.05), or trending towards significant (0.05 < *P* < 0.10), flux during incubation in Day 12 (D12; *n* = 5) and Day 16 (D16; *n* = 4) uterine lumen fluid (ULF). Predicted and unknown metabolites are excluded. Scaled intensities of select metabolites are provided, wherein the central horizontal line represents the median value with outer boundaries depicting upper and lower quartile limits. Error bars depict the minimum and maximum distributions, with a simple cross representing the mean value and an open circle the extreme data point. Corresponding statistical comparisons are also provided, wherein dark green shading indicates a significant (*P* ≤ 0.05) decrease (metabolite ratio < 1.0) between groups, whereas light green depicts a decreasing trend (0.05 < *P* < 0.10). Conversely, dark red shading indicates a significant (*P* ≤ 0.05) increase (metabolite ratio > 1.0) between groups, with pink depicting an increasing trend (0.05 < *P* < 0.10). Gray cells indicate the mean fold-change value was not significantly different for that comparison.

Thereafter, to probe the specific metabolites underpinning these findings across all subjects, we analyzed the data *en masse* and identified 33 metabolites exhibiting (*P* ≤ 0.05) or trending (0.05 < *P* ≤ 0.10) towards exhibiting significant flux by day (Fig. 3). More specifically—despite the aforementioned inter-subject ULF variation, some overlap was observed, as—the concentrations of 4 metabolites differed, and 10 trended towards a difference during the incubation of ULF collected on Day 12 (*n* = 5). Correspondingly, the levels of 13 metabolites differed, and 8 trended towards differing during the incubation of ULF on Day 16 (*n* = 4). In other words, these differences were statistically uniform across all heifers on each given day. Thus, different metabolic pathways appear active in ULF on Day 12 (expected day of conceptus elongation onset) as compared to Day 16 (expected day of maternal pregnancy recognition), as previously suggested by pseudotime analysis. Interestingly, regarding the statistically significant directionality of this metabolite flux, some trajectories were uniform [e.g., 3-methylcytidine; (iso)butyrate; nicotinamide riboside; and spermine], whereas others fluctuated (e.g., glutarate; methylsuccinate; and ophthalmate). In the future, reducing ULF incubation intervals while increasing the overall incubation duration may shed light on broader metabolite flux trends at play.

**Fig. 4:**
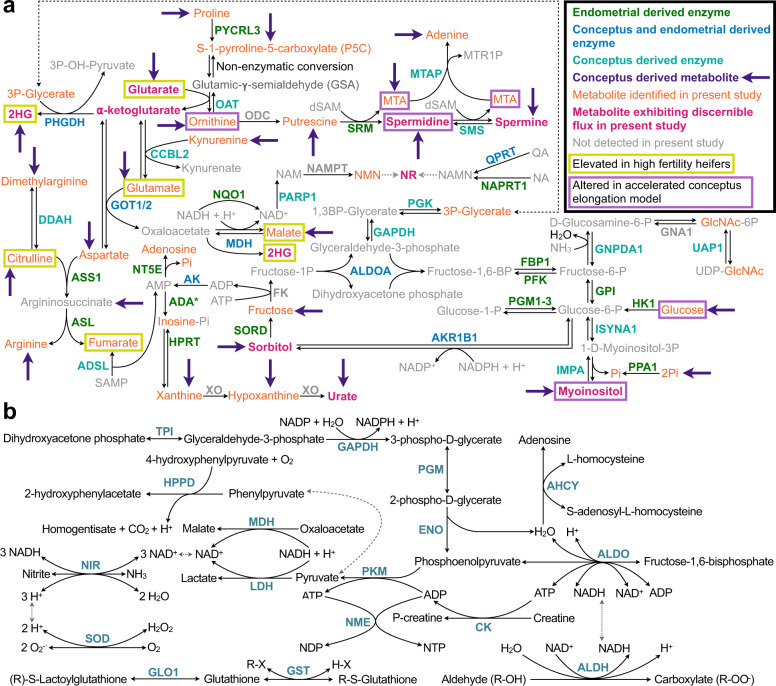
Predicted mechanism underpinning the metabolic semi-autonomy of uterine lumen fluid. **a** Extrapolated pathways active in bovine uterine lumen fluid (ULF) based on: (i) ULF proteomic data^19^; (ii) conceptus conditioned culture medium proteomic^24^ and metabolomic^13^ data—representing potentially active pathways during pregnancy; (iii) ULF metabolomic data presented in this study; (iv) ULF metabolomics from high vs. low fertility heifers^20^; and ULF metabolomic profiling from heifers with high systemic P4 (a model of accelerated conceptus elongation)^18^. **b** Predicted pathways active in human ULF based on existing proteomic data^21^. Abbreviations: 2-hydroxygluratare (2HG); 5′-methylthioadenosine (MTA); 5-methythioribose-1- phosphate (MTR1P); adenosine tri- (ATP), di- (ADP), and mono- (AMP) phosphate; decarboxylated s-adenosyl methionine (dSAM); inorganic phosphate (Pi); N-acetylglucosamine (GlcNAc); nicotinic acid (NA); nicotinic acid mononucleotide (NAMN); nicotinamide mononucleotide (NMN); nicotinamide (NAM) adenine dinucleotide (NAD) phosphate (NADPH); nicotinamide riboside (NR); succinyl-adenosine monophosphate (SAMP); uridine diphosphate (UDP); and quinolinic acid (QA). Enzyme abbreviations are listed in the Human Genome Organization (HUGO) Gene Nomenclature Committee database (genenames.org).

Next, to piece together the precise reactions underpinning the observed metabolic semi-autonomy of ULF, these data were cross-referenced against prior interrogations of the ULF proteome. Thereafter, to probe whether this predicted mechanism of ULF metabolic semi-autonomy may have physiological relevance (i.e., contribute to maternal-embryo dialogue), we conducted an integrative analysis, incorporating previous ULF metabolomic, as well as conceptus-conditioned media proteomic and metabolomic, data, as cumulatively presented in Fig. 4a and described below.

Moraes et al. previously probed the proteome and primary metabolome of ULF from embryo-transferred fertility-classed heifers on Day 17, and the ULF was obtained by flushing the uterus post-mortem^20^. Levels of 221 proteins differed in high (HF) vs. low (LF) fertility cattle—namely revolving around amino acid synthesis, vitamin B6 metabolism, and energy metabolism. Similarly, 70 (of 122 identified) metabolites differed in the ULF of HF vs. LF heifers. Interestingly, of the top 20 differentially abundant metabolites in HF vs. LF subjects, 30% correspond to metabolites and pathways predicted central to the semi-autonomous metabolic cascades of ULF (Fig. 4a). Another study examining the ULF metabolome on Days 12 and 14 from heifers with high systemic P4—a model of accelerated conceptus elongation—found 19 elevated metabolites in the ULF of high vs. normal P4 heifers^18^, of which 21% are common to the model of ULF semi-autonomy presented here (Fig. 4a). Moreover, an intimate relationship between conceptus-secreted metabolites^13^ and enzymes^24^ and the ULF metabolome data generated here is apparent (Fig. 4a), further supporting the idea that the metabolic semi-autonomy of ULF plays a role in uterine support of conceptus elongation and pregnancy establishment. Specific biochemical players, central to this phenomenon and previously identified as being of potential reproductive significance include glutarate, myo-inositol, sorbitol, spermidine, and spermine. Figure 4b highlights select biochemical reactions predicted to be similarly semi-autonomous in human ULF, based on published proteomic analyses^21^.

Regarding study limitations, retrieving ULF using smaller lavage volumes prior to analysis may improve both metabolomic analytical sensitivity and enzymatic proximity, to yield a higher-resolution atlas of active reactions. However, there is, unfortunately, no perfectly clean way to retrieve ULF. Here, we opted for direct retrieval by lavage, as opposed to in situ cannulation or *post-mortem* excision and flushing, as it presents a lower risk of sample deviation from the in vivo environment^29^. Cannulation of the uterine lumen would cause local inflammation and contamination. Spontaneous metabolite thermal degradation could also skew the data, particularly as metabolites in this study were underivatized. However, we do not expect this to be significant as glutamate, a notoriously unstable metabolite^30^ remains intact in solution for over 60 min at 60 °C^31^. The longest incubation duration in the present study was 20 min at 38 °C. Residual cellular, a cellular organelle, or blood contamination in the ULF was minimized in the present study as (a) visibly blood-contaminated ULF flushes were not utilized in this study (see Methods); (b) ULF aliquots were incubated in a closed system (Fig. 1e, f); and (c) low protein levels were present in all samples included for analysis (Fig. 1d).

Future work includes (a) similarly confirming the existence of this phenomenon in women, as predicted (Fig. 4b), and (b) interrogating the hypothesis that the degree of metabolic autonomy of ULF is linked to fertility. The latter could be used to develop biomarkers of fertility, and interventions to restore inadequate endometrial function. In closing, these data show that ULF biochemistry is semi-autonomously dynamic. Using cattle as a model, we demonstrate that select biochemical pathways are active within ULF, due to enzymatic activity. This finding, within the context of existing literature, enhances our understanding of the biochemical mechanisms leading to pregnancy establishment, with implications for improving fertility in domestic animals and women.

## Methods

### Animals

All animal procedures were conducted in accordance with the Guide for the Care and Use of Agriculture Animals in Research and Teaching and approved by the Institutional Animal Care and Use Committee at the University of Missouri. Fifteen (15) Holstein heifers were used averaging 15 months of age with a mean (±S.D.) body condition score (scale of 1–5) of 3.04 ± 0.44 and 766.6 ± 62.8 kg weight. On a random day of their estrous cycle, heifers were synchronized to estrus using an established protocol^32^. As depicted in Fig. 1a, heifers initially received a dose of gonadotropin-releasing hormone, coupled to the insertion of a controlled internal drug release (CIDR) intravaginal P4 insert (1.9 g, CIDR, Zoetis, NJ, USA). CIDR removal occurred 6 days thereafter, at which time, a PGF_2α_ dose (25 mg Dinoprost, Lutalyse, Zoetis, NJ, USA) was also administered, and heat detection patches (Estrotect, TN, USA) were applied. A second PGF_2α_ dose was administered 24 h later and heifers were visually observed for standing estrus at 12 h intervals for 72 h. Estrus was designated by heat detection patch activation.

Thereafter, heifers were distributed in two experimental groups for ULF retrieval on Days 12 and 16 post-estrus (Fig. 1a). On the day of ULF retrieval, the presence of an ovarian *corpus luteum* was confirmed by transrectal ultrasonography prior to uterine lavage^33^. Specifically, 10 ml PBS was gently expelled into the uterine body using a silicone catheter coupled to a syringe. The uterine body was gently massaged transrectally, and ULF was recovered by generating a mild negative pressure using the syringe. Upon retrieval, ULF was immediately processed as described below. All flushes were performed by the same technician. Of the 15 heifers initially enrolled, two were excluded on account of not exhibiting signs of estrus. The remaining 4 were removed, as ULF flushes were visibly contaminated with blood based on color.

### Uterine lavage collection and processing

Uterine lavages were immediately equally apportioned into 7 aliquots (Fig. 1b). All but two aliquots from each lavage were incubated at 38 °C with shaking (Genie Temp Shaker 100; Scientific Industries; Bohemia, NY, USA) for either 2, 5, 10, 15, or 20 min. Following incubation, these aliquots were immediately placed at 4 °C to effectively quench enzymatic activity and then clarified by centrifugation at 1000×*g* for 15 min at 4 °C (5424R; Eppendorf, Hamburg, Germany). One aliquot (Time 0) was maintained at 4 °C for 20 min prior to centrifugation as described. The supernatants were submerged in N_2_(*l*) and stored at −80 °C until shipment for analysis on dry ice, as described below. A “control” aliquot (Fig. 1b) was not processed and immediately flash frozen in N_2_(*l*) and stored at −80 °C until total protein was quantified (Fig. 1d) using a Qubit 3.0 Fluorometer (ThermoFisher, Waltham, MA, USA) as per manufacturer instructions.

### Progesterone quantification

To measure systemic progesterone (Fig. 1c), blood was withdrawn by coccygeal venipuncture into 10 ml vacutainer tubes (Fisher Scientific, Hampton, NH, USA) after uterine lavage and centrifuged at 1500 × *g* for 20 min at 4 °C. The supernatant (serum) was recovered and stored at −80 °C until analysis. Serum progesterone was quantified by double-antibody radioimmunoassay (MP Biomedicals, California, USA)^34^. The intra-assay coefficient of variation was 4% and assay sensitivity was 0.05 ng ml^−1^.

### Metabolomic profiling

Metabolomic analyses were performed by ultrahigh performance liquid chromatography–tandem mass spectroscopy (UPLC–MS/MS) by Metabolon Inc. (Durham, NC, USA)^13,18,35^. Briefly, the protein was precipitated and extracted using the automated MicroLab STAR system (Hamilton Company) with methanol under vigorous centrifugation at 680 × *g* for 2 min (Geno/Grinder 2000, Glen Mills) prior to methanol removal by TurboVap (Zymark) and overnight incubation in N_2_. Each sample was subsequently divided into four fractions—two for analysis by reverse-phase (RP) UPLC–MS/MS with positive ion mode electrospray ionization (ESI), one for analysis by RP UPLC–MS/MS with negative four-ion mode ESI, and one for analysis by hydrophilic interaction liquid chromatography (HILIC) UPLC–MS/MS with negative ion mode ESI. Sample extracts were then dried and reconstituted in solvents as outlined below.

The first fraction, analyzed under positive ionization, was subject to gradient elution (Waters UPLC BEH 1.7 μm C18 column 2.1 ×100 mm) in water and methanol with 0.05% perfluoropentanoic acid and 0.1% formic acid. The second fraction, run under positive ESI, was identically eluted, using the same column, but with an elution buffer additionally comprising acetonitrile. The third fraction, analyzed under negative ionization, was also eluted by a gradient buffer comprising methanol, water, and 6.5 mM ammonium bicarbonate (pH 10.8). The last fraction, ran under negative ESI, was eluted using a HILIC (Waters UPLC BEH Amide 1.7 μm column 2.1 ×150 mm) with a water and acetonitrile plus 10 mM ammonium formate (pH 10.8) gradient.

Samples were analyzed using a Waters Acquity UPLC coupled to a Thermo Scientific Q-Exactive high-resolution MS interfaced with heated electrospray ionization (HES-II) source and Orbitrap mass analyzer operating at 35,000 mass resolution and with a scan range between 70 and 1000 *m*/*z*. Metabolites were quantified against known internal and recovery standards, run in parallel at random intervals. Identification was based on retention time and an *m*/*z* within ±10 ppm. The technical (instrument) median relative standard deviation was 5% with a total process variability of 10%.

Three controls were analyzed in parallel with the experimental samples: (i) a pooled aliquot of all experimental samples, serving as a technical replicate control; (ii) ultra-pure water samples served as process blanks, also run in between the experimental samples at defined intervals; and (iii) a cocktail of quality control metabolites, absent from endogenous compound measurements, were spiked into each sample. The latter internal standard enabled instrument performance monitoring and chromatographic alignment.

### Metabolomic data extraction and analyses

Data were corrected for variations resulting from instrument inter-day tuning differences; median peak areas for each metabolite were registered as 1.00 prior to the proportional normalization and logarithmic transformation of each data point. For qualitative metabolomic analyses (e.g., Fig. 2), these data (Supplementary Data 1) were used (i.e., individual metabolite presence and/or flux, within the ULF of single heifers, were analyzed, with neither imputation nor statistical analysis). For semi-quantitative metabolomic interpretation (e.g., Fig. 3), missing values, if any, were imputed with the minimum observed value for each compound (Supplementary Data 2) prior to quantification by relative abundance using MetaboLync pathway analysis software (portal.metabolon.com), wherein statistical comparisons were made by two-way ANOVA with a *P* ≤ 0.05 or 0.05 < *P* < 0.10 cut off.

### Pseudotime analysis

Pseudotime analysis (Fig. 2w, x) was performed using partition-based graph abstraction, as in Wolf et al.^36^, to produce uniform manifold approximation and projection for dimension reduction projections in K-nearest neighbors space. More specifically, the Leiden Cluster Determination algorithm was applied to the relative concentration of all metabolites (*n* = 324) at each time point (*n* = 6), from the ULF of all heifers [Day 12 (*n* = 5) and 16 (*n* = 4)]. In summary, 9720 (Day 12) and 7776 (Day 16) data points were condensed to 9 and 10 clusters, respectively. Cluster trajectories (pseudotimes) were visualized by applying the ForceAtlas2 (FA) algorithm.

### Integrative metabolomic analyses

To predict the precise reactions underpinning the semi-autonomous metabolic nature of ULF (Fig. 4a), existing published datasets (cited below), in addition to Supplementary Data 1 and 2, were utilized. Specifically, endometrial-derived enzymes in ULF were gleaned from Forde et al.^19^, whereas conceptus and endometrial-derived enzymes were determined from Forde et al.^24^. Enzymes were considered conceptus-derived if present in conceptus-conditioned media^24^ but not in ULF from cyclic (non-pregnant) heifers^19^. Metabolites elevated in the ULF of high-fertility heifers vs. those of low fertility was established by Moraes et al.^20^, whereas metabolites elevated in the ULF of heifers with high systemic P4 (a model of accelerated conceptus elongation) are presented in Simintiras et al.^18^. Conceptus-conditioned media metabolites were identified in Simintiras et al.^13^. Human ULF proteomic data (Fig. 4b) were gleaned from DeSouza et al.^21^.

### Statistics and reproducibility

Serum progesterone (Fig. 1c) and ULF protein (Fig. 1e) comparisons were conducted by unpaired t-test using Prism 8 (GraphPad, San Diego, CA, USA). Total metabolite flux data (Fig. 1f-g) were visualized and statistically contrasted by two-way ANOVA coupled to Tukey’s non-parametric post hoc using Prism 8. Semi-quantitative individual metabolite flux (Supplementary Data 1) visualizations per subject (Fig. 2e-f) and per metabolite (Fig. 2g-t) were also achieved using Prism 8, as was heatmap (Fig. 2u) generation. Principal component analysis (Fig. 2v) was performed using the open-access Past4 software^37^. To elucidate common trends in metabolite flux, metabolomic data (Supplementary Data 2) were compared by two-way ANOVA, with *P* ≤ 0.05 denoting significance and 0.05 < *P* < 0.10 highlighting a trend toward significance. Metabolite flux was determined when a significant or trending difference was observed in ≥1 time-course comparison within a corresponding day (Supplementary Data 2; Fig. 3). All measurements were taken from distinct samples.

### Reporting summary

Further information on research design is available in the Nature Research Reporting Summary linked to this article.

## Supplementary information

Description of Additional Supplementary Files

Supplementary Data 1

Supplementary Data 2

Reporting Summary

## Data Availability

All data used to generate figures are provided with this paper and/or associated supplementary material. The raw metabolomic data are deposited in Dryad (datadryad.org) under Simintiras et al.^38^. Any additional information may be available from the corresponding author upon reasonable request.

## Code availability

The code used for pseudotime analysis is available on GitHub via https://bit.ly/3JpaLXD.
