# Supplementary material for: Uterine lumen fluid is metabolically semi-autonomous
Source: Commun Biol. 2022 Mar 1;5:191. doi: 10.1038/s42003-022-03134-0 (PMC8888695; doi:10.1038/s42003-022-03134-0)
Supplement: Supplementary file 1 — Description of Additional Supplementary Files [file 42003_2022_3134_MOESM1_ESM.pdf]

**Description of Additional Supplementary Files**

**File name:** Supplementary Data 1

**Description:** *Metabolomic profiling of uterine lumen fluid – individual heifer values*. Relative concentrations (scaled intensities) of all metabolites from the uterine luminal fluid (ULF) of each heifer at each duration of incubation, in addition to the phosphate buffered saline (PBS) blank control. Also provided are the relative concentration fold changes between select group comparisons. Cells are shaded using conditional formatting to highlight value magnitudes. Asterisks denote predicted metabolites.

**File name:** Supplementary Data 2

**Description:** *Metabolomic profiling of uterine lumen fluid – mean processed data*. Relative concentration fold changes (RCFC) of all metabolites from the uterine lumen fluid (ULF) from each heifer group, at each duration of incubation, following scaling, imputation, and statistical comparison by two-way ANOVA. Intra-day (*e.g.*, Time 5 *vs*. 2) and inter-day (*i.e.*, Day 12 *vs*. 16) comparisons are presented, wherein dark green shading indicates a significant ($P \leq 0.05$) decrease (metabolite ratio <1.0) between groups shown, whereas light green depicts a decreasing trend ($0.05 < P < 0.10$). Conversely, dark red shading indicates a significant ($P \leq 0.05$) increase (metabolite ratio >1.0) between groups shown with light red depicting an increasing trend ($0.05 < P < 0.10$). Non-colored cells and text indicate the mean fold-change value was not significantly different for that comparison. Also presented are all mean values with which comparisons were made, in addition to the percentage of filled values (*i.e.*, degree of imputation for that cohort). Technical parameters – such as metabolite Kyoto Encyclopedia of Genes and Genomes (KEGG), Human Metabolome Database (HMDB), Chemical Abstracts Service (CAS), and Refractive Index (RI) values – are also listed. Asterisks denote predicted metabolites.
