# Supplementary material for: Uterine lumen fluid is metabolically semi-autonomous
Source: Commun Biol. 2022 Mar 1;5:191. doi: 10.1038/s42003-022-03134-0 (PMC8888695; doi:10.1038/s42003-022-03134-0)
Supplement: Supplementary file 4 — Reporting Summary [file 42003_2022_3134_MOESM4_ESM.pdf]

nature portfolio

Corresponding author(s): Dr. Thomas E. Spencer

Last updated by author(s): Dec 28, 2021

Reporting Summary

Nature Portfolio wishes to improve the reproducibility of the work that we publish. This form provides structure for consistency and transparency in reporting. For further information on Nature Portfolio policies, see our Editorial Policies and the Editorial Policy Checklist.

Statistics

For all statistical analyses, confirm that the following items are present in the figure legend, table legend, main text, or Methods section.

n/a	Confirmed	
☐	☒	The exact sample size (*n*) for each experimental group/condition, given as a discrete number and unit of measurement
☐	☒	A statement on whether measurements were taken from distinct samples or whether the same sample was measured repeatedly
☐	☒	The statistical test(s) used AND whether they are one- or two-sided<br>*Only common tests should be described solely by name; describe more complex techniques in the Methods section.*
☐	☒	A description of all covariates tested
☒	☐	A description of any assumptions or corrections, such as tests of normality and adjustment for multiple comparisons
☐	☒	A full description of the statistical parameters including central tendency (e.g. means) or other basic estimates (e.g. regression coefficient) AND variation (e.g. standard deviation) or associated estimates of uncertainty (e.g. confidence intervals)
☐	☒	For null hypothesis testing, the test statistic (e.g. F, t, r) with confidence intervals, effect sizes, degrees of freedom and P value noted<br>*Give P values as exact values whenever suitable.*
☒	☐	For Bayesian analysis, information on the choice of priors and Markov chain Monte Carlo settings
☒	☐	For hierarchical and complex designs, identification of the appropriate level for tests and full reporting of outcomes
☒	☐	Estimates of effect sizes (e.g. Cohen's *d*, Pearson's *r*), indicating how they were calculated

Our web collection on statistics for biologists contains articles on many of the points above.

Software and code

Policy information about availability of computer code

Data collection	No software was used to collect data
Data analysis	Graphpad Prism 8.0 (heatmapping and statistical tests); Past4 (principal component analyses); and Scanpy v. 1.9 (pseudotime analysis). The code used for pseudotime analysis is available at https://github.com/hliu5259/singlecell/tree/master/Pseudotime

For manuscripts utilizing custom algorithms or software that are central to the research but not yet described in published literature, software must be made available to editors and reviewers. We strongly encourage code deposition in a community repository (e.g. GitHub). See the Nature Portfolio guidelines for submitting code & software for further information.

Data

Policy information about availability of data

All manuscripts must include a data availability statement. This statement should provide the following information, where applicable:

- Accession codes, unique identifiers, or web links for publicly available datasets
- A description of any restrictions on data availability
- For clinical datasets or third party data, please ensure that the statement adheres to our policy

All source data are provided with the manuscript and associated supplementary material, or are publicly available, as referenced in the manuscript. Moreover, the raw metabolomic data generated have been deposited to Dryad. Any additional information is available from the corresponding author upon reasonable request.

March 2021

Field-specific reporting

Please select the one below that is the best fit for your research. If you are not sure, read the appropriate sections before making your selection.

[x] Life sciences [ ] Behavioural & social sciences [ ] Ecological, evolutionary & environmental sciences

For a reference copy of the document with all sections, see nature.com/documents/nr-reporting-summary-flat.pdf

Life sciences study design

All studies must disclose on these points even when the disclosure is negative.

Sample size	Sample size was determined based on our previous experience in bovine uterine fluid metabolomic analyses.
Data exclusions	No data were excluded from the analysis
Replication	All replication attempts were successful. Each result described is based on at least four independent biological replicates.
Randomization	Cattle were randomly assigned to a synchronization protocol.
Blinding	The investigators were not blinded to group allocation due to the nature of this study.

Reporting for specific materials, systems and methods

We require information from authors about some types of materials, experimental systems and methods used in many studies. Here, indicate whether each material, system or method listed is relevant to your study. If you are not sure if a list item applies to your research, read the appropriate section before selecting a response.

Materials & experimental systems

n/a	Involved in the study	
[x]	[ ]	Antibodies
[x]	[ ]	Eukaryotic cell lines
[x]	[ ]	Palaeontology and archaeology
[ ]	[x]	Animals and other organisms
[x]	[ ]	Human research participants
[x]	[ ]	Clinical data
[x]	[ ]	Dual use research of concern

Methods

n/a	Involved in the study	
[x]	[ ]	ChIP-seq
[x]	[ ]	Flow cytometry
[x]	[ ]	MRI-based neuroimaging

Animals and other organisms

Policy information about studies involving animals; ARRIVE guidelines recommended for reporting animal research

Laboratory animals	This study did not involve laboratory animals
Wild animals	This study did not involve wild animals
Field-collected samples	All cattle used were housed at the University of Missouri Research Farm for the duration of this study under standard conditions.
Ethics oversight	All procedures using cattle were conducted in accordance with the Guide for the Care and Use of Agriculture Animals in Research and Teaching and approved by the Institutional Animal Care and Use Committee at the University of Missouri.

Note that full information on the approval of the study protocol must also be provided in the manuscript.
